# Identification of* Centella asiatica*'s Effective Ingredients for Inducing the Neuronal Differentiation

**DOI:** 10.1155/2016/9634750

**Published:** 2016-06-30

**Authors:** Hui Jiang, Guoshuai Zheng, Junwei Lv, Heyu Chen, Jinjin Lin, Yiyang Li, Guorong Fan, Xianting Ding

**Affiliations:** ^1^School of Biomedical Engineering, Institute for Personalized Medicine, Shanghai Jiao Tong University, Shanghai 200030, China; ^2^School of Pharmacy, Department of Pharmaceutical Analysis, Second Military Medical University, Shanghai 200433, China

## Abstract

*Centella asiatica*, commonly known as Gotu kola, has been widely used as a traditional herb for decades. Yet, the study on which compounds or compound combinations actually lead to its brain benefits remains scarce. To study the neuroprotection effects of* Centella asiatica*, neuronal differentiation of PC12 cells was applied. In our pilot study, we isolated 45* Centella asiatica* fractions and tested their abilities for inducing neuronal differentiation on PC12 cells. The most effective fraction showed robust induction in neurite outgrowth and neurofilament expression. LC-MS fingerprint analysis of this fraction revealed asiatic acid and madecassic acid as the dominant components. A further investigation on the pure combination of these two compounds indicated that the combination of these two compounds extensively promoted nerve differentiation* in vitro*. Application of PD98059, a protein MEK inhibitor, attenuated combination-induced neurofilament expression, indicating the combination-induced nerve differentiation through activation of MEK signaling pathway. Our results support the use of combination of asiatic acid and madecassic acid as an effective mean to intervene neurodegenerative diseases in which neurotrophin deficiency is involved.

## 1. Introduction

Neurotrophins are defined as molecules that regulate the survival, maintenance, and proliferation of specific neuronal subpopulations. These signaling molecules exert considerable control over the switch between life and death pathways in cells [1]. Decline of neurotrophic factors is often associated with disease pathology and symptoms [2]. However, peptidyl compounds hardly cross the blood-brain barrier in clinical studies due to their large molecular weight [3]. The administration of such protein-involving therapies in brain continuously shows limitations or failures in clinics [4]. A promising alternative treatment is to use neurotrophic factor-like small molecule agents.

*Centella asiatica*, commonly known as Gotu kola, is a medicinal plant from Apiaceae family native to Southeast Asian countries such as India, China, and Malaysia [5, 6]. Due to its medical importance, it is cultivated and used in ayurvedic medicine and traditional Chinese medicine (TCM) for centuries [6].* Centella asiatica* has been reported to promote wound healing [7–10], enhance the memory [11], decrease the inflammation [12, 13], and improve cognitive performance [13–16].* Centella asiatica* contains many constituents, such as asiatic acid, madecassic acid (6-hydroxy-asiatic acid), asiaticoside, madecassoside, betulinic acid, thankunic acid, and isothankunic acid [17]. In the past several decades, extensive efforts have been made to study the anti-inflammatory and would healing effects of these components. However, few reports are currently available on which of these constituents and/or constituent combinations contribute to brain benefits.

Here, we examined multiple ingredients and small molecules isolated from* Centella asiatica* for their neuroprotective effects. The neurotrophic activities of these ingredients in inducing neurite outgrowth and neurofilament expression were investigated. In addition, possible signaling pathways involved in the induction of neuronal differentiation were also unveiled. The neurotrophic factors identified from our results could serve as potential therapy for treating multifarious neurodegenerative diseases such as Parkinson's, Alzheimer's, and Huntington's diseases.

## 2. Materials and Methods

### 2.1. Materials

Asiatic acid and madecassic acid were purchased from Shanghai Yuanye Bio-Technology Co., Ltd. Absolute ethanol and methanol were purchased from Sinopharm Chemical Reagent Co., Ltd., China. All these reagents are of analytical reagent grade. All of the other chemicals were purchased from Sigma-Aldrich (St. Louis, MO, USA) except where noted. Millipore water (18.2 MΩ·cm) was used in the preparations of all aqueous solutions.

### 2.2. Preparation of* Centella asiatica* Extract

#### 2.2.1. Extraction of* Centella asiatica*

Dried* Centella asiatica* was purchased from Tongrentang (Beijing TRT, Beijing, China).* Centella asiatica* was first grounded into powder using a milling machine. 200 g* Centella asiatica* powder was eluted in 2 L 75% ethanol (Sinopharm Chemical Reagent, Shanghai, China) with continuous ultrasound for 1 h. This operation was repeated twice and the extractions were filtered and pooled together. The extraction was then evaporated to 400 mL concentrated liquid on a rotary evaporator (Shanghai SENCO Technology Co. Ltd., Shanghai, China) at 70°C.

#### 2.2.2. Fractionation of* Centella asiatica*

The extracted fluid was further separated into fractions using the macroporous resin method. The extracted fluid was loaded on a AB-8 macroporous resin column (200 g, 40 × 70 mm) and sat statically for an hour. The column was eluted at the rate of 20 mL/min with multiple solvents in the following sequence: 0.9 L water, 1.5 L 20% aqueous ethanol, 1.2 L 50% aqueous ethanol, and 0.9 L 75% aqueous ethanol. After the elution started, every 100 mL distillate was collected within a 100 mL conical flask. When the above elution finished, 45 fractions were collected in total. These fractions were further evaporated to 10 mL, separately. In the following parts of this paper, extractions are labeled according to the following rules: A-B, where A stands for alcohol concentration of eluant and B stands for the eluting sequence number with eluant A.

### 2.3. Cell Culture

Pheochromocytoma PC12 cells, a cell line derived from rat adrenal medulla, were maintained in Dulbecco's modified Eagle's medium (DMEM) containing 9% fetal calf serum (FBS), 6% horse serum (HS), 100 units/mL penicillin, and 100 *μ*g/mL streptomycin at 37°C in a humidified incubator with 5% CO_2_. Subcultures were digested with 0.25% trypsin when the cells reached 70% confluence. Cell culture medium was refreshed every other day. All culture reagents were purchased from Invitrogen Technologies (Carlsbad, CA, USA).

During the treatment with fractions, the cell culture medium was changed to DMEM containing 1% FBS, 1% HS, 100 units/mL penicillin, and 100 *μ*g/mL streptomycin for 3 h serum deprivation, and then cultured PC12 cells were treated with different reagents for 72 h. Cells treated with NGF (Alomone Laboratories, Jerusalem, Israel) at 50 ng/mL were used as positive control in this paper unless otherwise noted. In analyzing the signaling pathway, the cells were pretreated with MEK inhibitor PD98059 at 20 *μ*m for 5 h and then cotreated with different reagent for 72 h.

### 2.4. Cell Counting Kit-8 (CCK-8) Assay

Cell viability was assessed by using Cell Counting Kit-8 (Dojindo, Shanghai, China). PC12 cells were seeded in the 96-well plate at a density of 1.5 × 10^4^ cells per well and incubated for 24 h. The cells were then treated with the* Centella asiatica* fractions or other chemicals for another 72 h. CCK solution was added to the cell culture medium at a ratio of 1 : 10 and incubated for 2 h at 37°C. Absorbance of light with wavelength of 450 nm was recorded by an automatic detector (Biotek Synergy 2).

### 2.5. Neurite Stretch Measurement

The determination of neurite outgrowth in cell cultures was performed according to the previously published protocol [18]. After being treated with fractions, single compounds, or compound combinations for designed time, cells were fixed with ice-cold 4% paraformaldehyde for 30 min. Cultured PC12 cells from duplicate wells were photographed under a Leica microscope (Diagnostic Instruments, Sterling Heights, MI, USA), equipped with a phase-contrast condenser (20x objective lens). The microscope was connected to a digital camera, which captured and recorded 20 fields per well. Imaging J software was used to analyze the neurite presence and neurite length. For each treatment, at least 50 cells from 20 randomly chosen visual fields were analyzed and the neurite length readout was based on the calculation of these 1000 randomly examined cells. A PC12 cell was considered “differentiated” if one or more of its neurites were longer than the diameter of the cell body and further classified into three categories according to the length of its neurite: <15 *μ*m (or barely differentiated), 15–30 *μ*m (or moderately differentiated), and >30 *μ*m (or extensively differentiated).

### 2.6. Western Blot Analysis

PC12 cells were harvested and lysed in RIPA buffer (Millipore, CA, USA) containing Complete Protease Inhibitor Cocktail (Roche Diagnostics, Mannheim, Germany) with 1 mM PMSF (Sinopharm Chemical Reagent, Shanghai, China). After quantifications of the protein samples using BCA Protein Assay Kit (Pierce Biotechnology, Rockford, IL, USA), samples and controls were resolved on the 6% SDS-polyacrylamide gels and then wet electrotransferred to 0.45 *μ*m nitrocellulose membranes (Millipore, CA, USA). The membrane was marked (with pencil or India Ink) for identification and blocked with 5% fat-free milk freshly made in TBST on a rotating shaker for 1 h at room temperature. The primary antibodies used were antineurofilament 200 (NF200; Sigma, St. Louis, MO, USA), anti-NF68 (Sigma), and antiglyceraldehyde 3-phosphate dehydrogenase (GAPDH; Proteintech, Wuhan, China). The primary antibodies diluted in 10% BSA were added and incubated at 4°C overnight. The membrane was washed with TBST three times for 10 min, incubated with horseradish peroxidase- (HRP-) conjugated anti-mouse secondary antibody (Proteintech, Wuhan, China) at room temperature for 1 h, washed three times for 10 min with TBST, and detected by an ECL detection system (Pierce Biotechnology, Rockford, IL, USA). The intensities of the bands were quantified by densitometry using Gel-Pro Analyzer.

### 2.7. LC-MS/MS Instrument and Conditions

A Dionex UltiMate 3000 HPLC system and a ThermoFisher LCQFLEET mass spectrometer were employed to study the chemical compositions of the* Centella asiatica* fractions. A 20 *μ*L aliquot of plasma extract was injected into a YMC C18 column (250 mm × 4.6 mm, 5 *μ*m) at a column temperature of 25°C with a mobile phase composed of acetonitrile-water. The solvent gradient program was shown in Table 1. The column is at a flow rate of 1.0 mL/min with the split ratio of 3 : 7 flowing 300 *μ*L into the mass spectrometer. The analysis time was 60 min per sample, and the UV wavelength is 205 nm. The mobile phase was filtered through a 0.45 *μ*m nylon membrane filter before use. The temperature of the autosampler was kept at 4°C. The mass spectrometer was operated in negative ionization mode.

The LCQ mass spectrometer was operated with the capillary temperature at 350°C, sheath gas at 40 (arbitrary units), and the auxiliary gas at 9 (arbitrary units). The electrospray voltage was set to 4 kV, the capillary voltage at −45 V, and the tube lens offset at −95 V. Mass spectra were recorded from* m/z* 100–1300 at unit mass resolution without in-source fragmentation. For sequential MS^*n*^ experiments, the normalized collision energy ranged from 35% with wideband activation turned off.

### 2.8. Statistical Analyses

All data are presented as mean ± SEM. Data were assessed by one-way ANOVA, followed by Student-Newman-Keuls* post hoc* test. *p* values less than 0.05 were considered statistically significant.

## 3. Results 

### 3.1. Effective Fractions on the Differentiation of PC12 Cells

**Figure 1:**
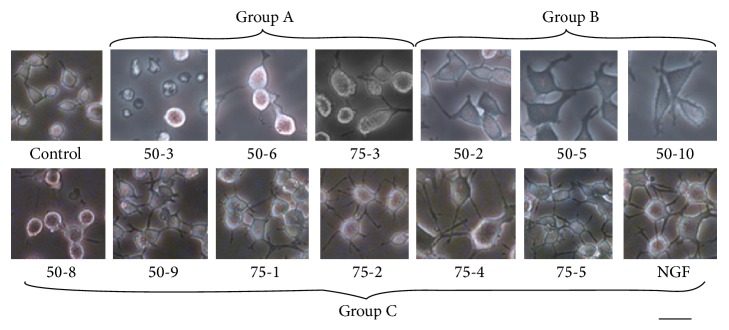
The fractions show wide range of neuronal inductive effects. After the PC12 cells were treated with NGF (50 ng/mL) and fractions (at the same dilution ratio of 1 : 50) for 72 h, randomly selected fields were observed using a camera attached to a microscope (×20). Control (A) 50-3, 50-6, and 75-3; (B) 50-2, 50-5, and 50-10; (C) 50-8, 50-9, 75-1, 75-2, 75-4, 75-5, and NGF. Bar = 50 *μ*m.

**Figure 2:**
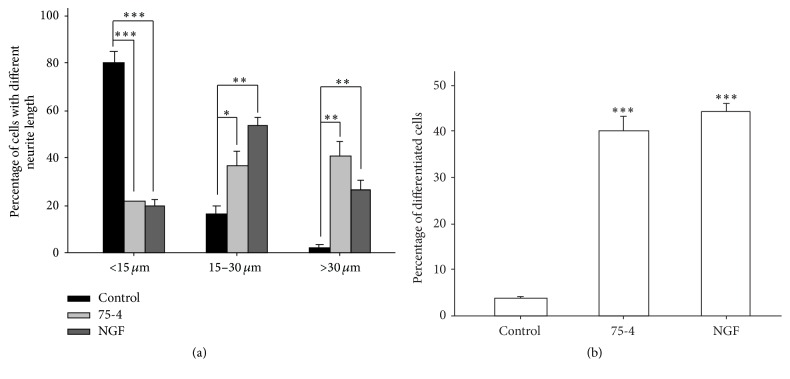
75-4 induces the neurite outgrowth of cultured PC12 cells. 75-4 (0.216 mg/mL) was applied into cultured PC12 cells for 72 h, with fresh medium or 75-4 every 24 h. NGF (50 ng/mL) served as the positive control. Cells were fixed with ice-cold 4% paraformaldehyde, and then the neurite outgrowth was examined under microscope. To quantify the differentiation effect, length of neurite (a) and the percentage of differentiated cell numbers (b) were counted as described in Materials and Methods. Data are expressed as the percentage of cells in 100 counted cells, mean ± SEM, *n* = 5, pooled from five independent experiments. Statistical comparison was made with the control; ^*∗*^ *p* < 0.05; ^*∗∗*^ *p* < 0.01; ^*∗∗∗*^ *p* < 0.001.

Forty-five fractions eluted by using the aforementioned method were screened for the potential neuronal differentiation effect. Cultured PC12 cells were treated with these fractions at the dilution ratio of 1 : 50 achieved from the CCK assay (Supplementary Figure in Supplementary Material available online at http://dx.doi.org/10.1155/2016/9634750). Then, fractions were divided into group A, group B, and group C according to the status of PC12 cells. They, respectively, appeared barely differentiated, moderately differentiated, and extensively differentiated according to the status of PC12 cells (Figure 1). We found that most fractions eluted with water and 20% aqueous ethanol showed poor effect on the neuron differentiation. Effective fractions were 50-8 (eluted with 50% aqueous ethanol, the 8th extraction), 50-9 (eluted with 50% aqueous ethanol, the 9th extraction), 75-1 (eluted with 75% aqueous ethanol, the 1st extraction), 75-2 (eluted with 75% aqueous ethanol, the 2nd extraction), 75-4 (eluted with 75% aqueous ethanol, the 4th extraction), and 75-5 (eluted with 75% aqueous ethanol, the 5th extraction). Among those fractions that showed strong inductive effects, 75-4 was found to deliver the most evident effects in inducing the neurite outgrowth for PC12 cells. Quantification on neurite outgrowth length for each cell was then carried out. Particularly, the length of neurites after the treatment with 75-4 on PC12 cells for 72 h was measured. The number of cells possessing neurite length of 15–30 *μ*m was increased over 2.2 times, while the number of cells possessing neurite length of >30 *μ*m was promoted to over 16.6 times (Figure 2(a)). The differentiated cells accounted for about 40% of the total cells (i.e., a cell with neurites longer than its body) (Figure 2(b)).

**Figure 3:**
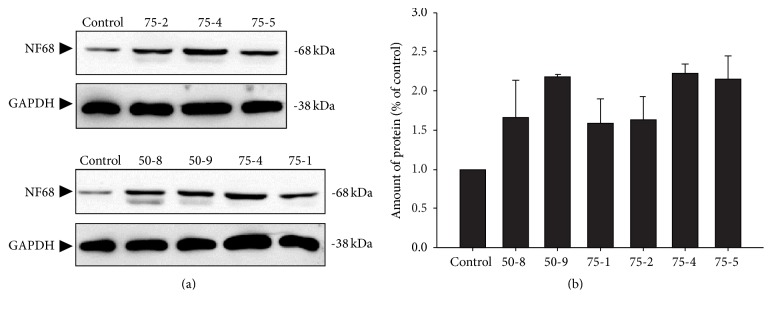
75-4 is the most effective fraction for inducing neurofilament expression of cultured PC12 cells. (a) Cultured PC12 cells were treated with 50-8, 50-9, 75-1, 75-2, and 75-4 at the same dilution ratio for 72 h. The cell lysates were collected to determine the expressions of NF68. NGF (50 ng/mL) served as the positive control. GAPDH served as loading control. (b) Quantification plot is shown in histograms. Values are expressed as the percentage of increase to basal reading (untreated culture), mean ± SEM. *n* = 5, pooled from five independent experiments.

In addition to the morphological exploration, the neurofilament expression in cultured PC12 cells under the treatment of strong inductive fractions, such as 50-8, 50-9, 75-1, 75-2, 75-4, and 75-5, was analyzed by Western Blot. NF68 is the most representative protein as a structural component of the differentiated neurons [18]. Our results indicated that after 72 h the treatment with these fractions induced the neurofilament expression. Among these fractions, 75-4 showed the most evident effect in inducing the expression of NF68 with a fold of ~225% (Figure 3). Therefore, 75-4 remarkably induces both neurofilament expression and neurite outgrowth.

### 3.2. LC-MS Analyses of 75-4 and 50-6

**Figure 4:**
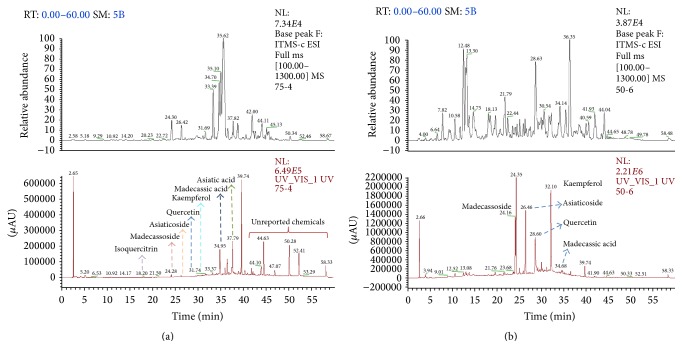
HPLC-UV and HPLC-MS^*n*^ analyses of 75-4 (a) and 50-6 (b) under optimized conditions (LC conditions: inject 20 *μ*L; column YMC C18 (250 mm × 4.6 mm, 5 *μ*m); column temperature 25°C; mobile phase: acetonitrile and water gradient the same as those in Table 1, flow rate of 1.0 mL/min; UV: 205 nm; LC-MS^*n*^ conditions the same as the previous ones, ionization: ESI neg; capillary temperature: 350°C; sheath gas: 40 (arbitrary units); auxiliary gas: 9 (arbitrary units); electrospray voltage: 4 kV; capillary voltage: −45 V; tube lens: −95 V).

On the basis of the aforementioned studies, 75-4 has been demonstrated to induce neuronal differentiation in terms of neurofilament expression and neurite outgrowth. Still, which active component accounts for the neuronal differentiation is less known. Then, we conducted LC-MS analysis of 75-4, and the result showed that 75-4 mainly contained six major chemicals (Figure 4(a)). However, except for asiatic acid and madecassic acid, no reports are available yet to determine what the other four chemicals are.

In this study, our LC-MS results indicated that these four chemicals were almost nonpolar and their molecular weights were about 277.63, 293.82, 309.98, and 279.70, respectively. Therefore, we inferred that they might be sesquiterpenes. Although asiaticoside, madecassoside, quercetin, isoquercitrin, and kaempferol have been detected to exist in 75-4 (Table 2), their percentages were much lower than those in the nonoptimal fraction 50-6 (Figure 4(b)).

### 3.3. Combination of Asiatic Acid and Madecassic Acid Induces the Neuronal Differentiation

Both asiatic acid and madecassic acid were found in 75-4 with high contents. Therefore, a natural question would be whether the combination of these two chemicals could also have synergistic effects on neuronal differentiation. The concentration of 75-4 we used in the aforementioned experiment was detected as 0.216 mg/mL after drying and weighing. Asiatic acid at 14.4 *μ*M, madecassic acid at 40.8 *μ*M (the amount in 0.216 mg/mL 75-4), and their combination were separately applied to PC12 cells for 72 h for inducing neurofilament expression.

**Figure 5:**
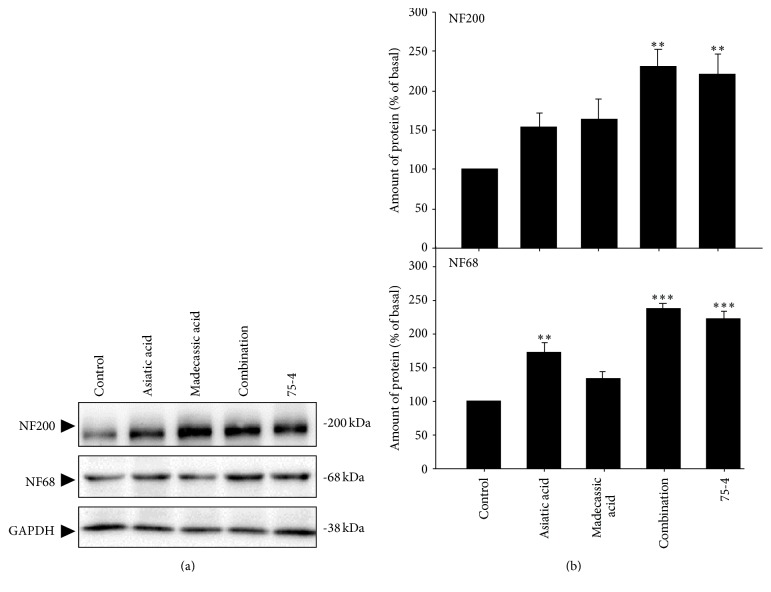
The combination of asiatic acid and madecassic acid found in 75-4 induces the expression of neurofilament of cultured PC12 cells. (a) Cultured PC12 cells were treated with asiatic acid (14.4 *μ*M), madecassic acid (40.8 *μ*M), their combination, and 75-4 (0.216 mg/mL) for 72 h. The cell lysates were collected to determine the expression of NF68 and NF200. NGF (50 ng/mL) served as the positive control. GAPDH served as loading control. (b) Quantification plot is shown in histograms. Data are expressed as the fold of change (100% of Basal) against the control, mean ± SEM, *n* = 4, pooled from four independent experiments. Statistical comparison was made between asiatic acid, the combination, and 75-4; ^*∗*^ *p* < 0.05; ^*∗∗*^ *p* < 0.01; ^*∗∗∗*^ *p* < 0.001.

As shown in Figure 5, asiatic acid induced the neurofilament expression, and madecassic acid showed moderate effects on neurofilament expression. However, the combination delivered superior induction on the expression of NF68 and NF200 compared to that of either individual chemical, with an increase by ~230% and 237%, respectively. This induction effect was similar to that of 75-4. These results therefore suggested the crucial role of asiatic acid and madecassic acid in neuron differentiation of 75-4.

### 3.4. The Effect of the Combination on MEK Signaling Pathway

**Figure 6:**
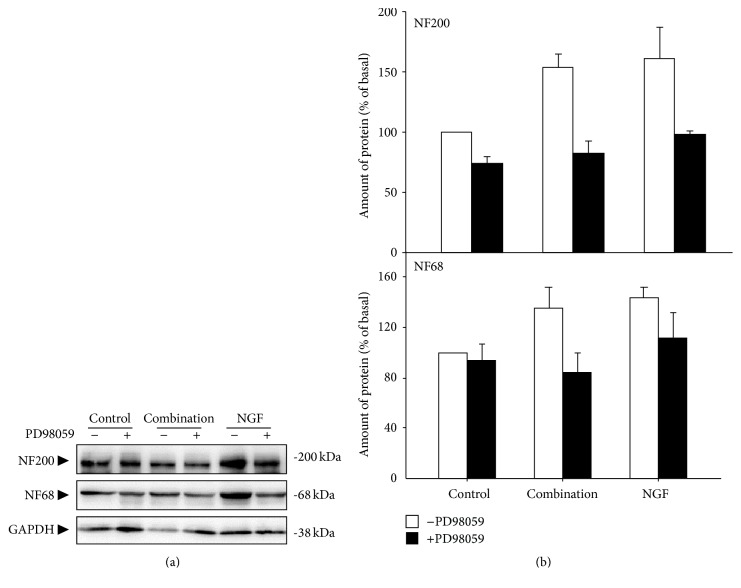
Inhibition of MEK signaling suppresses the combination-induced neurofilament expression of cultured PC12 cells. (a) Cultured PC12 cells were pretreated with or without MEK inhibitor PD98059 (20 *μ*m) for 5 h and then cotreated with the combination of asiatic acid (14.4 *μ*M) and madecassic acid (40.8 *μ*M) and NGF (50 ng/mL) for 72 h. The cell lysates were collected to determine the expression of NF68 and NF200. GAPDH served as loading control. (b) Quantification plot is shown in histograms. Data are expressed as the fold of change (100% of Basal) against the control, mean ± SEM, *n* = 4, pooled from four independent experiments.

Previous studies showed that inhibition of MEK can block the differentiation of PC12 cells induced by NGF [19]. To investigate whether MEK is involved in neuronal differentiation induced by the combination of asiatic acid and madecassic acid, PC12 cells were pretreated with PD98059, a selective MEK inhibitor, at 20 *μ*M for 5 h and then cotreated with (14.4 *μ*M) and madecassic acid (40.8 *μ*M) for 72 h. The NGF-induced neurofilament expressions were inhibited by PD98059 (Figure 6). Similarly, the combination-induced neurofilament expression could also be blocked by PD98059 (Figure 6). Our results suggested that the neuronal differentiation induced by the combination of asiatic acid and madecassic acid is, at least partially, due to the activation of MEK signaling pathway. This implied that the similarities existed in the differentiation mechanisms between the inductions triggered by the combination and NGF.

## 4. Discussion

Neuronal differentiation plays an initial and vital role in the development of neuron. Recently, morphological observation and biochemical detection as the main means of evaluating neuronal differentiation are generally reported. Cultured pheochromocytoma PC12 cell line is a widely employed model for studying neuronal differentiation in responding to various treatments. In this study, 45 fractions were screened for their differentiating effect on cultured PC12 cells and they showed wide range of neuronal inductive effects. Here, we found that 75-4 was the best fraction which showed significant inductive effect on both neurite outgrowth and neurofilament expression of NF68 and NF200. The expression of NF68 is a representative marker for the early stage of the neuron differentiation [20]. In contrast, the expression of NF200 is a marker for the late stage of neuron differentiation. The treatment of 75-4 in cultured PC12 cells could robustly stimulate the NF68 and NF200. Therefore, the involvement of 75-4 might support the whole neuron differentiation process.

As shown in the LC-MS profile, 75-4 contained a higher amount of asiatic acid, madecassic acid, and four other chemicals that have not been reported anywhere in other published literatures. The rest four chemicals that had molecular weights about 277.63, 293.82, 309.98, and 279.70, respectively, were almost nonpolar. Combined with their molecular weights and chemical properties, we speculated that they might be sesquiterpenes. They are currently under further investigations in our laboratory. Although asiaticoside, madecassoside, quercetin, isoquercitrin, and kaempferol have been detected to exist in 75-4 (Table 2), their percentages were much lower than those in the nonoptimal fraction 50-6 (Figure 4(b)). Since 75-4 showed superior neuronal differentiation ability compared to 50-6, the LC-MS fingerprint analysis between these two fractions suggested that the ingredients 75-4 contained were likely to be major substances leading to neuronal differentiation effects for* Centella asiatica*. In addition, other important and unreported chemicals might exist in* Centella asiatica*.

As asiatic acid and madecassic acid showed a relatively high amount in 75-4, we studied their neuronal differentiation effect. Here, we found that asiatic acid induced the neurofilament expression, which was in agreement with a previous report [21]. The fact that combination of them performs better than either chemical individually proves that the combination has the potential neurofilament expression synergy to better induce the neuron differentiation. No significant difference was found on the neurofilament expression level between 75-4 and the combination. The results strongly suggest that asiatic acid and madecassic acid play a vital role in neuronal differentiation, at least in cultured PC12 cells.

Nerve growth factor (NGF) is a secreted growth factor that plays an important role in the survival, maintenance, and regeneration of specific types of neurons in the central and peripheral nervous system. NGF was the first neurotrophin family member of nerve growth promoting factors. The “neurotrophic factor hypothesis” proposed that limited supplies of a target-derived neurotrophic factor can cause developing neuron death [22]. In accordance with this, NGF has been shown to be produced in target tissues of sensory and sympathetic fibres, taken up by the fibre terminals, and retrogradely transported back to the nerve cell body where it is required for the survival and maintenance of these neurons [2]. Many diseases of nervous system are related to NGF insufficiency, especially some neurodegenerative diseases [23], for example, depression [24] and Alzheimer's disease [25].

Therefore, the identification of neurite-promoting medicals and their combinations could be considered as a new direction in developing drugs or health food supplements. Asiatic acid and madecassic acid could potentially be used to develop new therapeutic agents. Furthermore, we demonstrate that the inhibition of MAP kinase could block the differentiation of PC12 cells induced by the combination and NGF, indicating that the combination of these two chemicals might lead to the neurite outgrowth through similar molecular mechanism as NGF. Taken together, the effective ingredients of* Centella asiatica* may be useful for the prevention and recovery of neurodegenerative diseases.

## 5. Conclusion

*Centella asiatica* is a tropical plant with multiple medical value. In this study, 75-4 has been found to induce the neurite outgrowth independently and display a striking effect on the increase of neurofilament expression. The LC-MS analysis has identified six chemicals as the major ingredients of 75-4, including asiatic acid, madecassic acid, and the other four chemicals that receive very limited discussions in both structure and biological properties among published literatures. Based on the expression of neurofilament, our results indicate that asiatic acid and madecassic acid are the main chemicals involved in the differentiation. In addition, our data also suggest that the combination of asiatic acid and madecassic acid induces neuronal differentiation partially through mediating MEK signaling way.

## Supplementary Material

At these concentrations, the fractions 50-5, 75-2, 75-4 and 75-5 had almost neither cytotoxicity nor proliferating while the fractions 50-2 and 50-5 showed relatively strong cytotoxicity to PC12 cells.

## Figures and Tables

**Table 1:** The solvent gradient program.

Time (min)	Water%	Acetonitrile%
0	95	5
25	65	35
50	0	100
55	0	100
55.1	95	5
60	95	5

**Table 2:** The identification results.

Rt (min)	[M-H]^−^	Fragments	Name
23.8	974.18	469, 323	Madecassoside
31.7	285.51	257, 151	Kaempferol
26	994.3	958.28, 469	Asiaticoside
34.8	503.8	457.33, 485.52	Madecassic acid
37.5	488.08	469.54, 409.4	Asiatic acid
28.3	301.5	179.04, 151.11	Quercetin
17.8	301	179, 151	Isoquercitrin
