# Supplementary material for: Identification of Centella asiatica's Effective Ingredients for Inducing the Neuronal Differentiation
Source: Evid Based Complement Alternat Med. 2016 Jun 30;2016:9634750. doi: 10.1155/2016/9634750 (PMC4944037; doi:10.1155/2016/9634750)
Supplement: Supplementary file 1 — At these concentrations, the fractions 50-5, 75-2, 75-4 and 75-5 had almost neither cytotoxicity nor proliferating while the fractions 50-2 and 50-5 showed relatively strong cytotoxicity to PC12 cells. [file 9634750.f1.pdf]

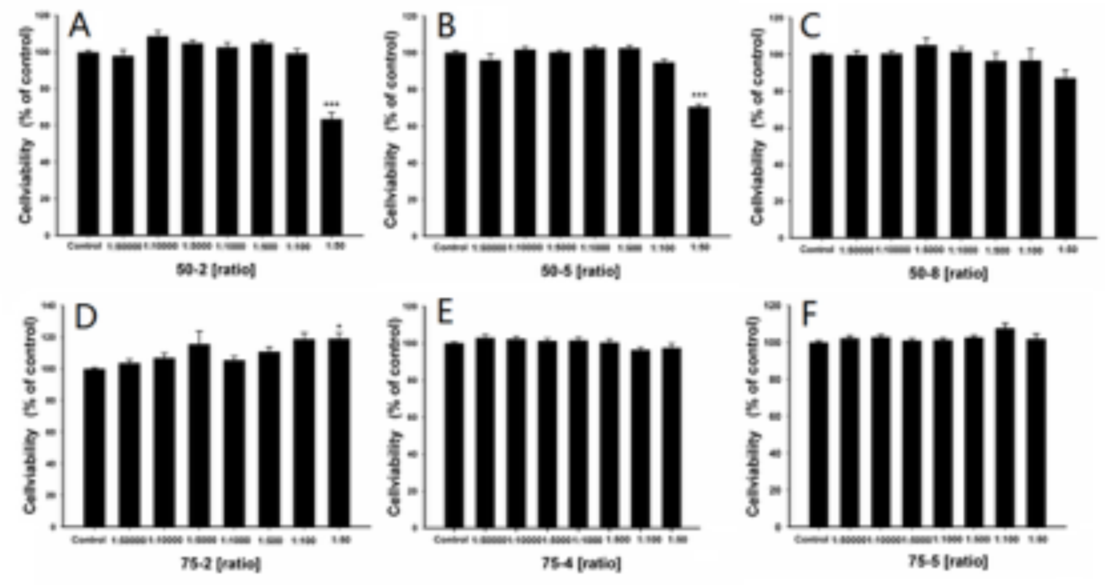

Supplementary figure: The cytotoxicity of representative fractions in PC12 cells

PC12 cells were seeded on to 96-well plate and incubated for 24 hours. After that, the cells were treated with 50-2, 50-5, 50-8, 75-2, 75-4,and 75-5 in different concentration for another 72 h. The CCK solution was added to the cell cultures and incubated for 1 hour at 37°C. Absorbance was measured at 450 nm in a microplate reader. Values are expressed as the percentage of total cells against the control , mean ± SEM, n = 4, pooled from four independent experiments, (*) $p < 0.05$; (**) $p < 0.01$; (***) $p < 0.001$.
